# Absence of *Bartonella bacilliformis* bacteraemia in asymptomatic individuals during an inter-outbreak period in an endemic Andean region

**DOI:** 10.1186/s12879-026-14195-5

**Published:** 2026-08-11

**Authors:** Helena Biasizzo, Palmira Ventosilla, César Ugarte-Gil, Nelson Gerardo Solórzano Espíritu, César V. Munayco, David A. J. Moore

**Affiliations:** 1 London School of Hygiene and Tropical Medicine, London, UK; 2 Instituto de Medicina Tropical Alexander von Humboldt, Universidad Peruana Cayetano Heredia, Lima, Perú; 3 Department of Epidemiology, The University of Texas Medical Branch, Galveston, TX USA; 4 Centro Médico La Esperanza, Caraz, Áncash, Perú; 5 Centro Nacional de Epidemiología, Prevención y Control de Enfermedades, Ministerio de Salud, Lima, Perú; 6 Laboratorio de Investigación de Enfermedades Infecciosas, Universidad Peruana Cayetano Heredia, Lima, Perú; 7 Present Address: Department of Infectious Diseases, General Hospital Celje, Celje, Slovenia

**Keywords:** *Bartonella bacilliformis*, Asymptomatic infection, Humans, Blood cultures, Inter-outbreak period, Reservoir

## Abstract

**Background:**

Carrión’s disease, caused by *Bartonella bacilliformis*, is endemic in the Andean valleys of Perú, Colombia, and Ecuador. The disease is believed to be transmitted by *Lutzomyia verrucarum* sandflies and typically occurs in seasonal outbreaks during periods of high vector activity. Although asymptomatic carriers are thought to maintain transmission between outbreaks, the presence of asymptomatic bacteraemia during inter-outbreak periods remains unclear.

**Methods:**

We conducted a cross-sectional study in two rural Andean communities in the Áncash department of Perú during the inter-outbreak season (July 2019). Participants ≥ 1 year of age without clinical signs of Carrión’s disease were enrolled. Demographic data, medical history, and physical examination were recorded, and venous blood samples were collected for the culture of *B. bacilliformis*.

**Results:**

Of 219 individuals invited, 197 provided samples; after exclusions for clotted or contaminated cultures, 177 were included in the analysis. The median age was 34 years (IQR, 25–49), and 64% of the participants were female. Eleven participants (6.2%) reported previous Oroya fever, and 50 (28.2%) had a history of verruga peruana. After 60 days of incubation, none of the 177 blood cultures were positive for *B. bacilliformis*. Thus, there were no asymptomatic bacteraemic individuals identified in our study during inter-outbreak period.

**Conclusions:**

In this study, we did not detect bacteraemic asymptomatic individuals during the inter-outbreak period. Further studies in different regions and seasons with more sensitive molecular methods are warranted to clarify the role of asymptomatic carriers in the epidemiology of Carrión’s disease.

**Supplementary Information:**

The online version contains supplementary material available at https://doi.org/10.1186/s12879-026-14195-5.

## Background

Carrión’s disease is a bacterial disease that affects principally communities in arid and semi-arid Andean valleys in Perú, Colombia and Ecuador, characteristically in geographical areas between 500 and 3200 m above sea level [1, 2].

It is caused principally by *Bartonella bacilliformis*, a Gram-negative coccobacillus with an affinity to invade erythrocytes and endothelial cells [3, 4]. The disease can manifest in two different clinical forms. The acute hematic form (Oroya fever) can range from a mild febrile illness to a life-threatening disease with haemolytic anaemia and multi-organ failure. The case-fatality rate of Oroya fever without antibiotic treatment can be as high as 88% [5–7]. The chronic eruptive form (verruga peruana) usually presents weeks to months after the acute form; however, it may also present without a preceding acute hematic illness [6]. It is characterised by wart-like skin lesions (verrugas), reminiscent of bacillary angiomatosis seen with other *Bartonella* spp. infections, sometimes accompanied by constitutional symptoms (fever, malaise, fatigue, weight loss) and arthralgias [4].

*B. bacilliformis* is believed to be transmitted from human to human by the bite of an infected sandfly of the genus *Lutzomyia*, in the majority of cases *Lutzomyia verrucarum* [8, 9]. The dynamics of disease transmission follows the seasonal activity of the putative sandfly vector, with the majority of acute cases present during the rainy season (December-May), while chronic cases are usually evenly spread during the year, with a peak of cases during dry months (May-September). Consequently, the outbreaks of the disease in the affected communities occur predominantly during the rainy season (the outbreak period), while there is a period during the dry season where there are no cases in the communities (the inter-outbreak period) [2, 10].

The majority of cases occur in Perú, especially in the Department of Áncash, which has the highest endemicity, followed by Huánuco and Cajamarca [4, 11]. However, the epidemiology of Carrión’s disease has changed in recent decades, attributed to the influence of climatic changes such as El Niño [12, 13]. It was hypothesised that alterations in environmental conditions associated with El Niño phenomenon act favourably on the propagation of the putative vector, with the emergence of cases in previously non-endemic geographic areas such as Amazonas and Cuzco and an increase in cases in endemic regions [13].

No animal reservoir has been identified, and it is believed that asymptomatic humans (bacteraemic individuals without Oroya fever or verruga peruana) represent the reservoir for *B. bacilliformis* during the inter-outbreak period, act as a bridge between the outbreaks in endemic communities and play an important role in the perpetuation of the disease [4, 14].

A small number of studies have reported that asymptomatic *B. bacilliformis* infection is frequent in endemic areas [14, 15]. According to the studies done during the outbreak period, blood culture-proven prevalence of asymptomatic bacteraemia in the population can range from 0 to 9.4%, whereas asymptomatic infection determined using polymerase chain reaction (PCR) yielded a prevalence of 0.5% [14–16]. It was proposed by Herrer in 1953 that asymptomatic carriage is present in endemic regions during the outbreak period, but gradually wanes with time [15]. However, according to our knowledge, there has been no previous study to determine the presence of asymptomatic humans with *B. bacilliformis* bacteraemia during an inter-outbreak period.

Detecting asymptomatic bacteraemic individuals can be challenging due to the absence of clinical signs/symptoms and low-level bacteraemia. Although no comparative studies exist, based on available literature, blood cultures appear to be a reliable method for the diagnosis of *B. bacilliformis* bacteremia in asymptomatic humans [17–19].

Our study aimed to investigate asymptomatic carriage during inter-outbreak period (without any concurrent cases of *B. bacilliformis* clinical forms) in endemic Andean communities in the Áncash department.

## Methods

### Study design and setting

**Fig. 1:**
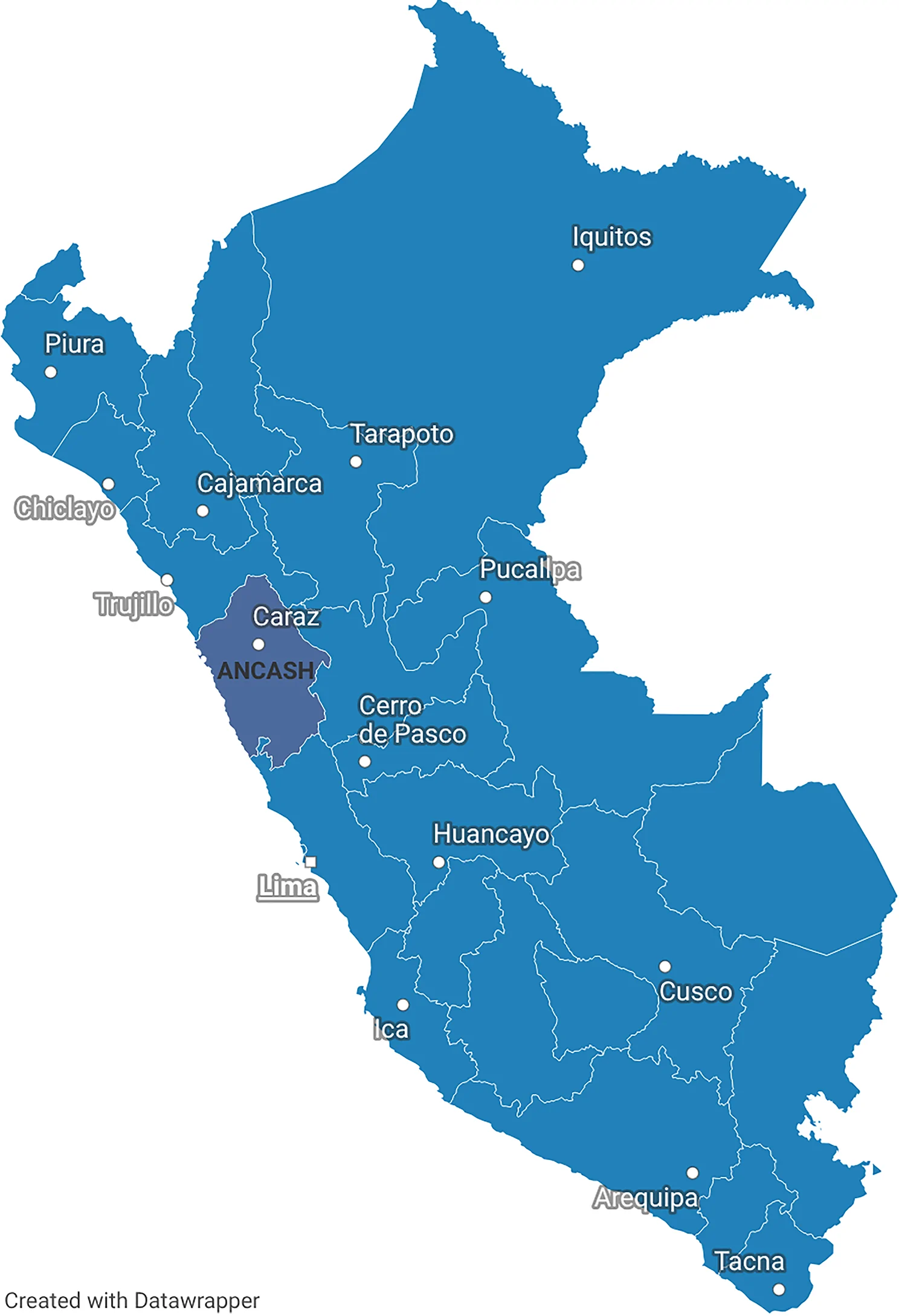
Figure of Perú showing the Áncash Department

**Fig. 2:**
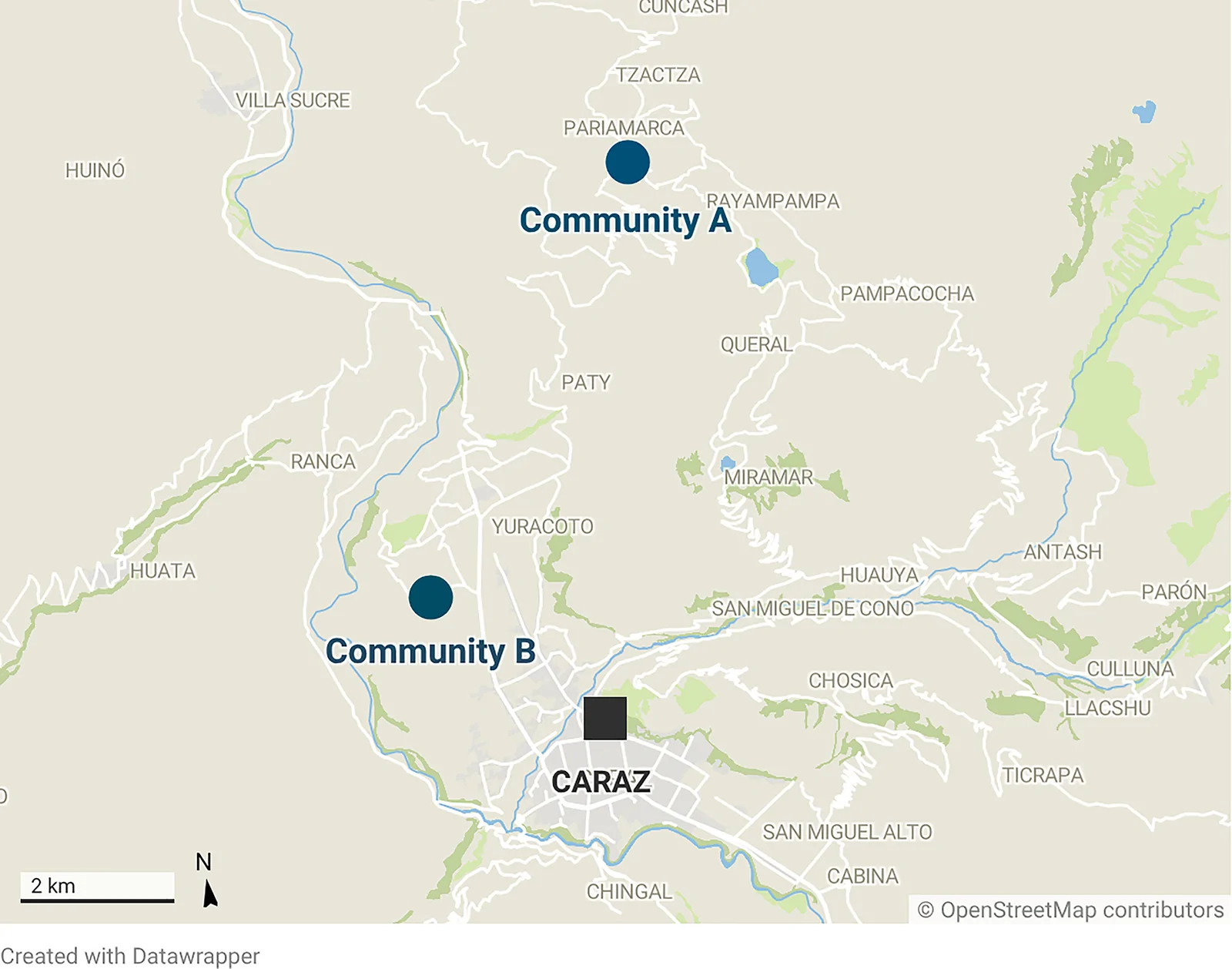
Detailed map showing both study locations in the Áncash Department

The study was cross-sectional, and recruitment took place in Áncash department in Perú between 24th and 30th July 2019 (during the inter-outbreak season). The inter-outbreak period was defined as the time during which local health services had not reported any active cases of Carrión’s disease (Oroya fever or verruga peruana). There was no active surveillance conducted in the communities, and case information relied solely on routine reporting to local health services rather than systematic case-finding. The study sites were selected in collaboration with the *Centro Nacional de Epidemiología*,* Prevención y Control de Enfermedades* of the Perú Ministry of Health in Lima and local Health Workers in Caraz as being highly representative of the endemic region for *B. bacilliformis* infection in Perú. Two communities with different recent exposure histories were included in the study: one community reported cases of disease within the preceding year, whilst the other had previously recorded cases, but none within the preceding year. The first community (A) had a population of 814 people and lies approximately 2700 m above sea level. The last cases of the disease (Oroya fever or verruga peruana) were seen in March 2019, four months earlier. The second community (B) had a population of 1318 and lies at an altitude of 2200 m. The distance between the study sites is 8 km. The most recent cases of the disease in community B were detected in March 2018, 16 months earlier. Both communities were rural, and the inhabitants were of Quechua-Spanish descent. The chief source of income in the region is agricultural activity. The study site is shown in detail in Figs. 1 and 2.

### Sample size and sampling strategy

The required sample size for estimating the proportion of asymptomatic *B. bacilliformis* bacteraemic individuals was calculated using the standard formula for proportions, $\:n={Z}^{2}p(1-p)/{d}^{2}$. We assumed an expected prevalence (p) of 5% based on the most rigorous previous study, a 95% confidence level (Z = 1.96), and a precision (d, margin of error) of ± 3% [15]. This calculation indicated that approximately 200 participants (n) were needed. We therefore recruited 100 individuals from each of the two study communities, yielding a total sample of approximately 200 participants.

During the preparation of the study, stratified random sampling was planned as a sampling method. We planned to stratify individuals in each chosen community by age group. Unfortunately, this was not feasible because reliable community-wide age data was not available as had been anticipated. Consequently, convenience sampling was used. Households that were readily accessible within the study communities were visited, and eligible individuals were enrolled sequentially. An indeterminate number of households was approached, and recruitment continued until the expected sample size was reached.

### Eligibility and exclusion criteria

Every person living in the endemic area older than one year was considered eligible for the study. Exclusion criteria were as follows: persons with clinical symptoms or signs of Oroya fever (fever, sweats, hepatomegaly, jaundice, lymphadenopathy, splenomegaly) or verruga peruana (warts, fever, arthralgias).

### Case definition

A case in this study was defined as an individual, currently living or a person who had lived in the endemic region in the past, without any concurrent clinical manifestation(s) of any form of Carrión’s disease and who had a positive *B. bacilliformis* blood culture.

### Data collection

Potential participants were approached with the help of local health workers and invited to take part in the study. After giving informed consent, a questionnaire was filled in with the help of the researcher and using Open Data Kit [20]. Next, clinical examination was performed, and finally, blood was collected from each participant.

The questionnaire was comprised of questions related to demographic data, self-reported medical history and possible previous treatment for Carrión’s disease (See Supplementary material).

Each participant was examined for possible signs or symptoms of acute (fever, sweats, hepatomegaly, jaundice, lymphadenopathy, splenomegaly) or eruptive form (warts, fever, arthralgias) of disease.

Blood was drawn from an antecubital vein using a Vacutainer system and aseptic technique. 6 ml of blood was drawn into a sodium citrate test tube. Blood samples were stored at room temperature in Áncash for 5 days, then transported to the Laboratory of Experimental Microbiology at Instituto de Medicina Tropical Alexander von Humboldt, Universidad Peruana Cayetano Heredia in Lima, where 6 ml of blood was immediately inoculated into slant blood culture bottles. The culture medium was composed of Columbia Blood Agar with 10% sheep blood and incubated at 29 °C for 60 days under ambiental CO_2_ levels in a humidified incubator.

Slant medium culture bottles were examined weekly for possible signs of *B. bacilliformis* growth or contamination with other bacteria. If bacterial growth was detected, subcultures were made. Bacterial colonies would be Gram-stained, and the DNA of those with morphology suggestive of *B. bacilliformis* would be tested using PCR. The details of the PCR protocol have been described previously [21].

### Statistics

The primary outcome of the study was asymptomatic *B. bacilliformis* bacteraemia, defined as a positive blood culture for *B. bacilliformis* in an asymptomatic individual.

Categorical data were summarised as percentages, and continuous variables were described using medians and interquartile ranges. Had positive blood cultures been detected, associations between categorical variables would have been assessed using Fisher’s exact test, with odds ratios and corresponding 95% confidence intervals calculated for exposures considered potential risk factors for asymptomatic bacteraemia. A significance level of *p* < 0.05 would have been applied.

### Ethics

The study received ethical approval from the relevant ethics committees of the London School of Hygiene & Tropical Medicine, Universidad Peruana Cayetano Heredia in Lima, and the Ministry of Health in Áncash, Perú. Written informed consent was obtained from all participants, or from a parent/legal guardian for those under 18 years of age. Participants were provided with a clear explanation of the study’s purpose, procedures, and potential risks and benefits. The study was conducted in accordance with the principles of the Declaration of Helsinki.

## Results

**Fig. 3:**
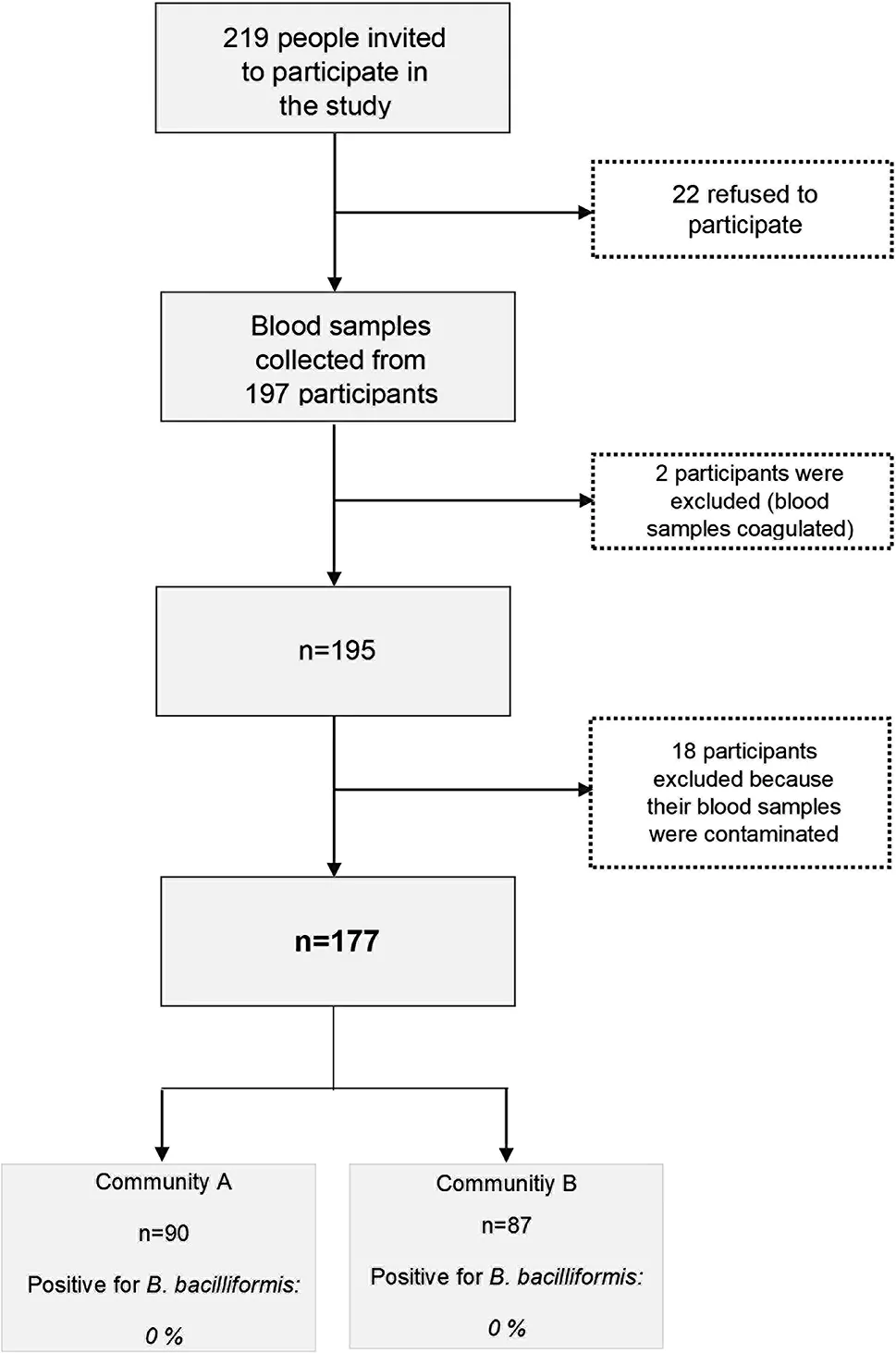
Study flow chart

219 people were invited to participate in the study from the 24th – 30th of July 2019. 22 of them declined participation for personal reasons, and therefore, blood samples were collected from 197 people. Two blood samples clotted before inoculation into blood culture bottles and were excluded from the study. An additional 18 blood cultures were contaminated with other bacteria and were also excluded from the analysis as being unevaluable. Altogether, 20 participants were excluded because of unusable blood samples, and the final sample was 177 participants, 90 from Community A and 87 from Community B (See Fig. 3).

### Demographic characteristics of the participants

The majority of people included in the study were females (64.4%), and the median age of the participants was 34 years (interquartile range 25–49). Two-thirds of participants were aged 18–55 years, and most worked in farming or were houseworkers. Demographic characteristics of study participants are presented in detail in Table 1.

**Table 1:** Demographic characteristics of the study participants

Characteristic	Community A *(N = 90)*	Community B *(N = 87)*	Total *(N = 177)*
	Number (%)	Number (%)	Number (%)
**Gender**			
*Male* *Female*	29 (32.2) 61(67.8)	34 (39) 53 (61)	63 (35.6) 114 (64.4)
**Age (years)**			
1–18 19–36 37–54 > 55	6 (6.7) 39 (43.3) 24 (26.7) 21 (23.3)	19 (21.8) 29 (33.3) 32 (36.8) 7 (8.1)	25 (14.2) 68 (38.4) 56 (31.6) 28 (15.8)
**Occupation**			
Farmer House worker Student Other	35 (39) 48 (53.3) 3 (3.3) 4 (4.4)	30 (34.5) 35 (40.2) 16 (18.4) 6 (6.9)	65 (36.7) 83 (46.9) 19 (10.7) 10 (5.7)

Out of 20 people excluded from the study because of unusable blood samples (2 coagulated samples and 18 contaminated blood cultures), 15 were females and 5 males, 10 of them were living in Community A and 10 in Community B. None of them reported having suffered with the Oroya fever in the past, but 5 of them did report having had verruga peruana (4 in community A and 1 in community B).

No potential participants were excluded based on exclusion criteria (clinical manifestations of the disease).

### Past Oroya fever

11 out of 177 people had been diagnosed with Oroya fever in the past, 3 from Community A and 8 from Community B. 10 of the 11 were 18 or less at the time of the diagnosis (the 11th was aged 22 years at the time). The time since diagnosis was a median of 16 years ago (interquartile range 14 to 45 years). All of them received treatment for the disease at the hospital.

### Past verruga peruana

Regarding the verruga peruana, 50 (28.2%) participants reported having manifestations consistent with the disease in the past. The median time since the diagnosis was 25 years (interquartile range 18 to 37 years). Thirty-eight of 50 (76%) were under 18 years of age at the time of diagnosis; the majority (66%) self-cured without having received any treatment for the disease.

None of the participants who were diagnosed with Oroya fever or verruga peruana in the past could name the antibiotic they were treated with.

Past history of Oroya fever and verruga peruana among participants is summarized in Table 2.

**Table 2:** Past history of Oroya fever and verruga peruana among study participants

Characteristic	Community A *(N = 90)*	Community B *(N = 87)*	Total *(N = 177)*
	Number (%)	Number (%)	Number (%)
**History of Oroya fever**			
Yes No	3 (3.3) 87 (96.7)	8 (9.2) 79 (90.8)	11 (6.2) 166 (93.8)
*Oroya fever present*	*N = 3*	*N = 8*	*N = 11*
*<18 years*	*3 (100)*	*7 (87.5)*	*10 (91)*
*≥ 18 years*	*0 (0)*	*1 (12.5)*	*1 (9)*
*Treated for Oroya fever*			
*Yes*	*3 (100)*	*8 (100)*	*11 (100)*
No	0 (0)	0 (0)	0 (0)
**History of verruga peruana**			
Yes No	30 (33.3%) 60 (66.7%)	20 (23) 67 (77)	50 (28.2) 127 (71.8)
*Verruga peruana present*	*N = 30*	*N = 20*	*N = 50*
*<18 years*	*22 (73)*	*16 (80)*	*38 (76)*
*≥ 18 years*	*8 (27)*	*4 (20)*	*12 (24)*
*Treated for verruga peruana*			
*Yes*	*8 (26.7)*	*9 (45)*	*17 (34)*
No	22 (73.3)	11 (55)	33 (66)

### Microbiological results

None of the blood cultures was positive after 60 days of incubation. Thus, there were no bacteraemic asymptomatic individuals detected in our study during the inter-outbreak period. Because no bacterial growth suggestive of *B. bacilliformis* was observed in any of the culture bottles, no PCR was performed. Contaminated cultures demonstrated growth with colony morphologies not characteristic of *B. bacilliformis*, thereby indicating contamination by non-*B. bacilliformis* organisms. Out of the contaminated excluded samples (*N* = 18), 7 grew Gram-positive cocci, 10 Gram-positive bacilli and one sample grew yeast.

## Discussion

This study was performed to investigate asymptomatic carriage of *B. bacilliformis* during the inter-outbreak period in an endemic area using blood cultures. Our research did not identify bacteraemic asymptomatic individuals.

Although viable *B. bacilliformis* can persist within verrucous lesions and individuals with verruga peruana may be present year-round in some endemic areas, many communities experience prolonged periods without any clinical cases of Carrión's disease. In such circumstances, if humans constitute the primary reservoir, the continued presence of *B. bacilliformis* in the community would require asymptomatic bacteraemic individuals. Other studies that also reported asymptomatic humans were done during the outbreak of Oroya fever or during the period when there were cases of symptomatic individuals present in the community [14, 15, 19]. In contrast, our study site and period were specifically chosen to avoid this situation. The study was conducted in two different communities in the endemic region with different recent experiences of the disease. One community had last detected cases of any form of Carrión’s disease 4 months prior, and the other community 16 months before the initiation of the study. Consistent with the results of other studies, most reports of previous disease, of either form (Oroya fever or verruga peruana), were in children [10]. None of the blood cultures grew *B. bacilliformis* after 60 days of inoculation. Our contamination rate of 9.1% is higher than the usually accepted contamination rate in conventional blood culture of 2–3% [22, 23] but comparable with rates of 7–20% in similar field studies of Carrión’s disease [24], reflecting the logistical challenges of sampling in remote, mountain locations.

In our study, we used blood cultures as a method for the detection of *B. bacilliformis* bacteraemia in asymptomatic individuals. While there are numerous methods available, research in this area is limited, and most studies compare detection methods for *B. bacilliformis* during Oroya fever, where bacterial concentration in the blood is higher [25]. Two forms of the disease with known lower bacterial concentrations in the blood—verruga peruana and asymptomatic form—pose a specific challenge for the diagnosis. In the only study that measured the prevalence of asymptomatic *B. bacilliformis* using PCR in an endemic region (finding it in 0.5%), comparison with other methods was not possible since only PCR was used [14]. Based on the available data, we prioritise blood cultures over PCR for the following reasons: First, blood cultures analyse a larger volume of blood compared to the PCR method. Second; blood cultures can detect viable bacteria, which have the potential to be transmitted to and replicate in another organism, compared to PCR, where non-viable DNA fragments of the bacteria that are already dead can be detected [17–19].

We did not consider blood smear a suitable test for the detection of asymptomatic infection, as the sensitivity of this method is very low [1, 26].

We acknowledge that additional serological or molecular diagnostic techniques would have strengthened the study. Enhanced detection methods, such as enrichment culture, molecular assays, and serial blood sampling, previously applied to identify other *Bartonella* spp. may increase the likelihood of detecting low-level or intermittent *B. bacilliformis* bacteremia. Even though molecular assay sensitivity varies with the amplification platform, target gene, and sample type, additional testing with quantitative PCR, nested PCR, and/or digital PCR could potentially detect *B. bacilliformis* DNA in blood from asymptomatic individuals who were negative by the culture-based method used in our study [27–30]. However, due to the limited financial resources this was not possible. Another factor that could have reduced the detection of *B. bacilliformis* in blood cultures is the unavoidable delay between blood collection and culture inoculation. Samples could only be processed upon arrival in Lima, resulting in a 5-day interval that may have decreased bacterial viability. This logistical limitation, inherent to conducting field work in remote settings, should also be considered when interpreting the absence of culture-detectable bacteraemia.

To increase the external validity of the study, simple random sampling of the communities with a community as a sampling unit should have been done. Because some of the communities were extremely remote and also due to limited time for the study, the communities were chosen with the help of local disease experts. The originally conceived study employed stratified random sampling so that the sample would be representative of the population, and the results of the study would be generalisable to the target population. However, any type of probability sampling was not feasible at the time of the study. Therefore, convenience sampling was used.

We are aware that non-probability sampling leads to selection bias and, therefore, we cannot conclude that the sample of our study is entirely representative of the target population. Nevertheless, we sampled a large number of people in two populations in which transmission had undoubtedly occurred in the past.

Another limitation of this study is the small number of participants under 18 years of age in our sample. Although bartonellosis is more common in children, recruiting minors was difficult because they were reluctant to provide blood samples. This reduced pediatric representation and should be considered when interpreting both age-specific patterns and the overall study results.

Data on the effectiveness of treatment for either Oroya fever or verruga peruana remain very limited. To date, only one study has directly compared therapeutic regimens for the verruga peruana, reporting that azithromycin achieved similar or slightly greater reductions in bacteraemia and lesion duration than rifampicin [31]. Beyond this, robust evidence on cure rates or long-term protection following treatment is lacking. Available epidemiological observations also suggest that immunity after infection is acquired, but may not be fully protective. During the Caraz district outbreak in 2003, described by Sánchez Clemente, several individuals reported multiple episodes of *B. bacilliformis* infection over previous years, indicating that reinfection is possible. That study further documented frequent under-dosing during routine clinical care, which may contribute to incomplete treatment responses and recurrent disease [10].

The results of our study suggest that bacteraemic asymptomatic individuals are not present in significant numbers in this endemic region during the inter-outbreak periods. This hypothesis was initially proposed by Herrer in 1953 when he was measuring serial culture positivity in a group of students who had recently come to live in an endemic area. It was concluded that asymptomatic bacteraemic humans failed to produce positive cultures on repeated testing [1, 15]. If we attempt to explain our findings in this context, asymptomatic *B. bacilliformis* infection may be present in significant numbers predominantly when there are clinical cases of the disease (during the rainy season) in the same community, and it wanes with time. To our knowledge, our study is the first to attempt to identify asymptomatic individuals with *B.* *bacilliformis* bacteraemia during the inter-outbreak season, with no detected clinical cases in the communities for some months before the start of the study.

## Conclusion

Our research did not demonstrate *B. bacilliformis* bacteraemia in asymptomatic individuals during the inter-outbreak period in endemic Andean communities under the study conditions. Further investigations, preferably longitudinal studies using highly sensitive detection methods that could capture temporal dynamics of *B.bacilliformis* transmission are warranted to gain a comprehensive understanding of the epidemiology of *B. bacilliformis* infection, encompassing diverse geographical regions, as variations in disease characteristics may exist beyond the confines of our current study area.

## Supplementary Information

Below is the link to the electronic supplementary material.

Supplementary Material 1

Supplementary Material 2

## Data Availability

The datasets used and/or analysed during the current study are available from the corresponding author on reasonable request.
