# Supplementary material for: Absence of Bartonella bacilliformis bacteraemia in asymptomatic individuals during an inter-outbreak period in an endemic Andean region
Source: BMC Infect Dis. 2026 Aug 11;26:1965. doi: 10.1186/s12879-026-14195-5 (PMC13746406; doi:10.1186/s12879-026-14195-5)
Supplement: Supplementary file 1 [file 12879_2026_14195_MOESM1_ESM.pdf]

<table>
<tr><td colspan="4">Bacteriemia por B. bacilliformis en individuos asintomáticos durante los períodos entre brotes, Ancash, Julio 2019</td><td>Fecha:</td></tr>
<tr><td rowspan="2">Datos generales</td><td>Comunidad:</td><td>Apellido:</td><td>Nombre:</td><td>Codigo paciente:</td></tr>
<tr><td>Edad:</td><td>Genero:
□ Femenino
□ Masculino</td><td></td><td>Ocupación:</td></tr>
<tr><td rowspan="2">Fiebre de la Oroya</td><td>Ha tenido Fiebre de la Oroya?
□ Si
□ No</td><td>Cuando?</td><td colspan="2">Toma de antibióticos?
□ Si
□ No</td></tr>
<tr><td>Antibiótico(s):
□ ciprofloxacina
□ ceftriaxona
□ azithromycina</td><td>□ penicillina
□ ampicillina
□ rifampicina</td><td>□ clindamycina
□ gentamycina
□ streptomycina</td><td>□ trimethoprima/sulfa
□ datos no disponibles
□ otro: ___________</td></tr>
<tr><td rowspan="2">Verruga peruana</td><td>Ha tenido verruga peruana?
□ Si
□ No</td><td>Cuando?</td><td colspan="2">Toma de antibióticos?
□ Si
□ No</td></tr>
<tr><td>Antibiótico(s):
□ ciprofloxacina
□ ceftriaxona
□ azithromycin</td><td>□ penicillina
□ ampicillina
□ rifampicina</td><td>□ clindamycina
□ gentamycina
□ streptomycina</td><td>□ trimethoprima/sulfa
□ datos no disponibles
□ otro: ___________</td></tr>
</table>

**Baseline data**	**Community:**	**Surname:**	**Name:**	**Participant's ID number:**
	**Age:**	**Sex:**<br>□ Female<br>□ Male		**Occupation:**
**Oroya fever**	**Oroya in the past?**<br>□ Yes<br>□ No	**When?**	**Treated?**<br>□ Yes<br>□ No	
	**Antibiotic:**<br>□ ciprofloxacin<br>□ ceftriaxone<br>□ azithromycin	□ penicillin<br>□ ampicillin<br>□ rifampicin	□ clindamycin<br>□ gentamycin<br>□ streptomycin	□ trimethoprim/sulfa<br>□ no data<br>□ other: __________
**Verruga peruana**	**Verruga in the past?**<br>□ Yes<br>□ No	**When?**	**Treated?**<br>□ Yes<br>□ No	
	**Antibiotic:**<br>□ ciprofloxacin<br>□ ceftriaxone<br>□ azithromycin	□ penicillin<br>□ ampicillin<br>□ rifampicin	□ clindamycin<br>□ gentamycin<br>□ streptomycin	□ trimethoprim/sulfa<br>□ no data<br>□ other: __________
